# Muscle-restricted knockout of connexin 43 and connexin 45 accelerates and improves locomotor recovery after contusion spinal cord injury

**DOI:** 10.3389/fphys.2024.1486691

**Published:** 2024-10-25

**Authors:** Carlos A. Toro, Rita De Gasperi, Katherine Vanselow, Lauren Harlow, Kaitlin Johnson, Abdurrahman Aslan, William A. Bauman, Christopher P. Cardozo, Zachary A. Graham

**Affiliations:** ^1^ Spinal Cord Damage Research Center, Bronx, NY, United States; ^2^ Department of Medicine, Icahn School of Medicine at Mount Sinai, New York, NY, United States; ^3^ Department of Phychiatry, Icahn School of Medicine at Mount Sinai, New York, NY, United States; ^4^ The Friedman Brain Institute, Icahn School of Medicine at Mount Sinai, New York, NY, United States; ^5^ Healthspan, Resilience and Performance, Florida Institute for Human and Machine Cognition, Pensacola, FL, United States; ^6^ Research Service, Birmingham VAHCS, Birmingham, AL, United States; ^7^ Department of Cell, Developmental, and Integrative Biology, University of Alabama at Birmingham, Birmingham, AL, United States

**Keywords:** spinal cord injury, Cx43, skeletal muscle-restricted knockout, Cx45, contusion spinal cord injury, transection spinal cord injury, recovery of function

## Abstract

Traumatic spinal cord injury (SCI) results in the disruption of physiological systems below the level of the spinal lesion. Connexin hemichannels (CxHCs) are membrane-bound, non-selective pore proteins that are lost in mature myofibers but reappear *de novo* on the sarcolemma after peripheral denervation, chronic SCI, diabetes, and severe systemic stress such as sepsis. Cx43 and Cx45 have been implicated as the major CxHCs present in diseased muscle, and muscle-restricted knockout of these genes reduces muscle atrophy after denervation, likely by reducing excess calcium influx with resultant inflammasome activation. A muscle-restricted Cx43/45 conditional knockout (mKO) mouse model was developed and tested to check whether it would improve outcomes following either a complete spinal cord transection at the level of thoracic vertebrae-9 (T9) or a motor-incomplete T9 impact-contusion SCI. mKO had no effect on the body mass after complete T9 transection. There was reduced atrophy of the plantaris 15 days post-SCI that was not associated with molecular markers of inflammation, hypertrophic/atrophic protein signaling, or protein and mRNA expression related to mitochondrial integrity and function. mKO mice had faster and greater locomotor recovery across 28 days after a motor-incomplete contusion SCI with no differences in spared white matter; male mKO mice generally had greater muscle mass than genotype controls post-injury, but muscle sparing was not observed in female mKO mice post-injury. The data establish a new paradigm where muscle Cx43/45 may contribute to the tissue crosstalk that determines the neuromuscular function of sub-lesional musculature after motor-incomplete SCI in a sex-dependent manner. Our novel findings should promote investigation to develop innovative treatment strategies to improve the function and quality of life for persons with SCI.

## Introduction

Spinal cord injury (SCI) is a devastating neurological injury that results in the loss of sensation and voluntary movement, with resultant bowel, bladder, and sexual dysfunction. Findings that SCI results in the rapid upregulation of hepatic expression of pro-inflammatory cytokines (Goodus et al., 2021) and that SCI results in a chronic low-grade increase in the levels of circulating cytokines in humans (Bank et al., 2015) suggest that the initial trauma and paralysis induce a systemic response that most likely impacts many tissues. Among the most significant changes observed after SCI are the rapid and extensive loss of skeletal muscle mass that, when combined with reduced physical activity, lowers the basal metabolic rate and, insulin-mediated glucose uptake (Bauman and Spungen, 2001). Skeletal muscle represents 40% of the mass of a healthy young adult and releases a wide range of mediators that modulate fat, bone, brain, and likely other tissues (Das et al., 2020; Pedersen and Febbraio, 2012). The profile of mediators released by skeletal muscle changes in response to exercise or immobilization. For example, our group has shown that paralysis from a sciatic nerve transection results in altered exosomal miRNA cargo from cultured myofibers (De Gasperi et al., 2017); however, the role of skeletal muscle in mediating the systemic effects of spinal cord injury remains poorly understood.

Muscle wasting is a highly coordinated and rapid biological process that involves catabolism of muscle proteins, which results in atrophy and loss of strength (Ebert et al., 2019; Bodine et al., 2023). Muscle wasting begins within a few days after the onset of trauma (e.g., burn), disease (e.g., sepsis), or disuse (e.g., paralysis) (Bodine, 1985; Graham et al., 2021). The paralysis caused by traumatic SCI results in one of the more severe forms of acute muscle wasting and extensive chronic muscle atrophy. Although complete anatomical spinal cord transections are rare in humans, loss of all voluntary movement below the anatomical level of SCI (motor-complete SCI) can occur despite there being some spared white matter at the injury site, and motor-complete SCI accounts for ∼30% of all cases (National Spinal Cord Injury Statistical Center, 2023). Most post-SCI neurological recovery occurs within the first 3 months of injury, with only a 5% conversion from motor-complete to motor-incomplete SCI from 1 year to 5 years post-injury (Kirshblum et al., 2004). Those with motor-complete SCI experience extensive atrophy of paralyzed muscles (Castro et al., 1999a; Castro et al., 1999b), loss of force-generating capacity (Castro et al., 1999b), and greater susceptibility to muscle damage after exertion (Bickel et al., 2004).

The devastating, life-long consequences of SCI highlight the need for novel translational medicine approaches to improve, and ultimately maintain, greater voluntary muscle function in those with SCI. Pharmaceutical interventions have showed little to no efficacy in preserving muscle mass and function after motor-complete SCI in pre-clinical models (Otzel et al., 2021), emphasizing the need to find novel mechanisms that guide future translational strategies.

Inhibition of connexin hemichannels (CxHCs) has recently been established as a promising direction to improve muscle health and function during muscle wasting (Saez et al., 2015). CxHCs allow the non-selective passage of small molecules into and out of the cell with excess calcium entry, intracellular calcium accumulation, and inflammasome activation being the best described pathological mechanistic processes (Saez et al., 2015; Plotkin et al., 2017; Giaume et al., 2013). CxHCs are important regulators for muscle differentiation (Araya et al., 2005) but are absent from the sarcolemma of healthy, mature fibers (Cea et al., 2013; Cisterna et al., 2020). However, they preferentially reappear *de novo* in fast-twitch myofibers in multiple models of diseases that induce muscle wasting. Conditional knockouts of Cx43 and Cx45 in skeletal muscle reduce denervation atrophy (Cea et al., 2013) and prevent the increased sarcolemmal permeability caused by denervation (Cea et al., 2013; Cisterna et al., 2020), endotoxemia (Cea et al., 2019), and diabetes (Cea et al., 2023). The effects of these genetic manipulations have been replicated by the oral administration of boldine (Cea et al., 2019; Cea et al., 2023; Burrell et al., 2023), which blocks open CxHCs (Yi et al., 2017; Toro et al., 2023).

Our group has found evidence that CxHC expression is likely to be altered after SCI. Specifically, we demonstrated *via* immunofluorescence staining that sarcolemmal Cx39, Cx43, and Cx45 protein expression are elevated in gastrocnemius muscles 56 days after a complete spinal cord transection in young male rats (Cea et al., 2013). We also showed in mice that boldine normalized at least some of the abnormalities of the skeletal muscle metabolome and transcriptome (Potter et al., 2023) and the circulating lipidome (Graham et al., 2023) 7 days post-complete spinal cord transection, but it did not prevent the atrophy of hind limb muscles or loss of body weight. Boldine administration also improved locomotor recovery following motor-incomplete SCI (Toro et al., 2023). No studies to date have tested the effects of conditionally regulating the expression of Cx43 and Cx45 on muscle biochemical, metabolic, or functional properties after SCI.

Our study aimed to determine whether skeletal muscle-restricted knockout (mKO) of Cx43 and Cx45 after SCI improves function, reduces muscle loss, or impacts molecular factors related to atrophy, inflammation, and mitochondrial dysfunction. We hypothesized that in a complete spinal cord transection model, mKO of Cx43 and Cx45 would not prevent muscle atrophy but would positively affect the biochemical and molecular properties of paralyzed muscles. Additionally, we predicted that unlike boldine, mKO would not enhance sensorimotor function after a motor-incomplete contusion SCI.

## Methods and materials

### Animals

#### Transgenic mouse development

All animal studies were approved by the IACUCs of the University of Alabama, Birmingham (approval #: 21639), and James J. Peters VAMC (approval #: CAR-16-54). We developed a skeletal muscle-restricted Cx43 and Cx45 knockout mouse using a Cre-loxP approach. First, commercially available mice with homozygous floxed *Gja1* (encoding Cx43) alleles [Cx43^(fl/fl)^; B6.129S7-I*Gja1* ^*tm1dlg*^/J, Jackson Laboratory, strain #008039] were bred with mice with homozygous floxed *Gjc1* (encoding Cx45) alleles [Cx45^(fl/fl)^] provided by Dr. James Nagy (Maxeiner et al., 2005), University of Manitoba, to generate Cx43^(fl/fl)^/Cx45^(fl/fl)^ mice. To generate the conditional knockouts, Cx43^(fl/fl)^/Cx45^(fl/fl)^ mice were bred with commercially available mice with a heterozygous Cre recombinase knock-in placed in the first exon of *Myod1* [MyoD-Cre^(+/−)^; FVB.Cg-*Myod1* ^*tm2.1(icre)Glh*^/J; Jackson Laboratory, strain #014140] to create a MyoD-Cre^(+/−)^-Cx43^(fl/fl)^/Cx45^(fl/fl)^ mouse, hereafter referred to as “mKO.” The genotype controls were littermates with no MyoD-Cre alleles, hereafter referred to as “Con”. Cx43^(fl/fl)^ and MyoD-Cre^(+/−)^ were chosen as they are commercially available and have been utilized successfully across multiple physiological domains; Cx45^(fl/fl)^ had no commercial vendor. MyoD-Cre^(+/−)^ was selected as it has been demonstrated to successfully activate Cre-loxP systems in a muscle-specific manner.

Genotyping was completed for all animals using genomic DNA isolated from ear snips using the QIAGEN Blood and Tissue kit (Cat. No. 69504) and an agarose gel electrophoresis system (Thermo Fisher Scientific). The primers used are detailed in Table 1.

**TABLE 1:** Primers used.

Gene	Forward sequence (5′-3′)	Reverse sequence (5′-3′)
Gja1	CTTTGACTCTGATTACAGAGCTTAA	GTCTCACTGTTACTTAACAGCTTGA
Gjc1	GGAAAGGCATATGTCACCACTCTTGGC	CTCTAGGAACACTGTAACCTGAGATGTCCC
MyoD-Cre	CGGCTACCCAAGGTGGAGAT	TGGGTCTCCAAAGAGACTCC
Mutated	GCGGATCCGAATTCGAAGTTCC	

#### Laminectomy, T9 spinal cord transection, and T9 spinal cord contusion surgeries

We have described our methods for laminectomy-only, T9 spinal cord transection, and T9 impact contusion injuries in detail previously (Toro et al., 2023; Graham et al., 2015; Graham et al., 2022). In brief, 4–8-month-old mKO and Con male and female mice received a laminectomy only (sham) or laminectomy, followed by spinal cord transection (tSCI) or 65 kdyne spinal cord impact contusion (cSCI). After measuring body mass, the mice were anesthetized with continuous inhalation of 2%–3% isoflurane. The hair around the spinal column was shaved using hair clippers, and the area was cleaned with 70% ethanol, followed by the topical application of betadine. An incision was made in the skin over T4–T11, and the spinal column was exposed by blunt dissection. The T9 vertebral arch was removed using fine forceps following cutting of the lateral sections with sharp surgical scissors to expose the dura. For mice in the sham group, the laminectomy site was promptly sutured in layers, with the incision site closed using wound clips. tSCI mice had the spinal cord cut completely using sharp surgical scissors. A probe was then passed through to ensure a complete transection, with a second cut used to sever the remaining spinal cord bridges as necessary. An inert gel foam was placed in the transection space to prevent spinal cord reattachment. For cSCI mice, after the laminectomy, they were placed within the lockable forceps of the Infinite Horizons impactor (Precision Systems and Instrumentation) under continuous isoflurane and received a 65-kdyne impact force. The force of 65 kdyne was chosen as we demonstrated that this severity results in temporary paralysis with acute muscle loss (Graham et al., 2022) with quantifiable longitudinal improvements in locomotor recovery (Toro et al., 2023). Following both transection and contusion SCI, the injury site was closed as noted above. All animals were placed in a clean cage with ALPHA-dri+ bedding and singly housed for the duration of the study, with wound clips being removed 10 days post-surgery.

In total, 106 mice were used for these studies. No sham mice prematurely died or were humanely euthanized before the study endpoint. Five out of 39 tSCI mice died (13%, 4F/1M) before the study endpoint: 3 (Con 2F; mKO 1F) from unknown post-operative complications within 24 h of surgery and 2 (mKO, 1M/1F) were humanely euthanized within the first 7 days due to excessive chewing of the hind limbs. For the cSCI mice, 17 out of 53 mice (32%; 8F/9M) died from post-operative complications or were humanely euthanized before the study endpoint. One mouse (Con, F) was euthanized immediately after impact contusion due to fractured vertebrae. Six mice were euthanized between 8 and 10 days after contusion due to sustained excessive body mass loss compared to pre-surgery (Con 3F; mKO 3F). Three mice were euthanized due to signs of excessive abdominal or hind limb chewing (Con 2F; mKO 1M). One mouse was euthanized due to a likely undetected bone hit during impact resulting in no locomotor recovery (mKO 1F). Lastly, six animals died during the first 72 h post-cSCI from unknown post-operative complications (two Con 1F/1F; four mKO 1M/3F). The final group sizes were Con-sham (n = 8, 4M/4F), mKO-sham (n = 6, 3M/3F), Con-tSCI (n = 19, 10M/9F), mKO-tSCI (n = 15, 4M/11F), Con-cSCI (n = 14, 8F/6M), and mKO-cSCI (n = 22, 10F/12M).

#### Post-operative care

All mice were placed in clean cages resting on 37°C recirculating water warming pads for 24 h post-surgery. They were subcutaneously administered a cocktail of Ketofen (5 mg/kg) and enrofloxacin (5 mg/kg) for 3 days, with additional volumes of lactated Ringer’s solution (up to 1 mL total per day) to maintain hydration. Bladders were manually expressed 2–3 times per day for the duration of the study.

#### Locomotor testing

We used the Basso Mouse Scale (BMS) (Basso et al., 2006) and mouse SCI-adapted horizontal ladder rung walking (Cummings et al., 2007) to test gross and fine motor function in cSCI mice, respectively, as described previously (Toro et al., 2023). In brief, the BMS test was performed in an open-field environment (1.5-m-diameter kiddie pool with custom plexiglass floor) by two trained investigators blinded to the experimental groups. After familiarization, measurements were taken pre-surgery and then at 3, 7, 10, 14, 21, and 28 days post-injury (dpi). The horizontal ladder rung walk (HLW) was tested using a 1-m-long ladder with a camera on a rail underneath to record all foot placements. The recordings were analyzed by a blinded investigator, with the negative stepping outcomes (hind paw drag, rung slip, and rung miss) counted and represented as a percentage of the total steps.

#### Euthanasia and tissue collection

The mice were euthanized for tissue collection by cardiac exsanguination, removal of the heart, and cervical dislocation at two different time points. Sham and tSCI mice were sacrificed 15 days post-SCI, with cSCI sacrificed 28 days post-SCI. Blood (200–500 µL) was placed in a 1.5-mL microcentrifuge tube to clot for 30 min and then spun at 2,000 rcf for 15 min at 4°C to collect serum. Hindlimb muscles [soleus, plantaris, gastrocnemius, tibialis anterior (TA), and extensor digitorum longus (EDL)], forelimb muscles (biceps and triceps), and the heart were carefully excised, weighed, and then flash-frozen in liquid nitrogen, with the exception of the left EDL, which was used for *ex vivo* contractile studies (described below). Lastly, a subset of n = 12 (n = 6 Con-cSCI and n = 6 mKO-cSCI) mice were subjected to transcardiac perfusion with PBS, followed by 4% PFA for whole-body fixation. Due to technical difficulties in animal and tissue processing, only n = 6 (n = 3 Con-cSCI and n = 3 mKO-cSCI) mice had samples that could be used for analyses.

#### EDL contractile function testing

The contractile function of the EDL from a subset of sham and tSCI mice was determined using the 1200A Isolated Mouse Muscle System from Aurora Scientific. 4–0 silk sutures were attached to the EDL at the proximal and distal tendons *in vivo*, with the muscle then removed and placed in a bath containing Krebs–Ringer solution (pH 7.4) supplemented with tubocurarine (30 µM) and glucose (11 mM) (Thermo Fisher Scientific, Cat. Nos J60222.MC and 15023021) with bubbling O_2_ at 25°C. The tendons were placed in the bath with the proximal tendon attached to the fixed hook in the bath and the distal tendon attached to the force transducer. The muscle was placed just under *in vivo* resting length and allowed to equilibrate for 5 min. The supraphysiological stimulation voltage was determined by stimulating the muscle with a single, 500-ms pulse at increasing voltages with 1-min rest until the force no longer increased. This value was then doubled. The optimal length (L_O_) was determined by lengthening the muscle in 0.5-mm increments, followed by a twitch stimulus with 1-min rest until the force plateaued. At L_O_, we tested for maximal twitch force, time-to-peak tension, and half-relaxation time. Muscles were allowed to rest for 5 min and then put through a force–frequency protocol using 300-ms pulses at 10, 25, 40, 60, 80, 100, and 150 Hz, with 1-min rest intervals between stimuli, with the muscle set back to L_O_ as necessary. Lastly, fatigue index was determined using 120 contractions delivered with a 300-ms pulse at 40 Hz and a 1-s rest interval between contractions. The fatigue index was calculated by dividing the force of the final contraction by the peak force generated during the protocol. All force data were analyzed using Dynamic Muscle Control Software (v5.5; Aurora Scientific).

#### Spinal cord histology

White and gray matter was measured using FluoroMyelin (Thermo Fisher Scientific, Cat. No. F34651) staining of immersion-fixed spinal cord tissue, as we have described (Toro et al., 2023). In brief, 10-µm sections were cut every 100 µm for 2 mm centered around the lesion using a Leica cryostat at −20°C. Images were captured using 20 × tiled images using a confocal microscope (Zeiss LSM 700) and quantified using ImageJ2 (version# 2.9.0/1.53t).

#### Serum and muscle multiplexed cytokine assay

Serum (1:1 v/v) and plantaris lysates (1:5 v/v) were used for cytokine detection using the Meso Scale Diagnostics V-PLEX Proinflammatory Panel 1 Mouse Kit (Cat. No. K15048D-2) following the manufacturer’s instructions. The plates were run using the Meso Scale QuickPlex SQ 120 system and analyzed using Meso Scale Discovery Workbench.

#### Skeletal muscle RNA isolation and RT-qPCR

RNA was isolated from 5–10 mg of the left plantaris muscles using the QIAGEN miRNeasy Tissue/Cell Advanced kit (Cat. No. 217684) according to the manufacturer’s instructions, with homogenization completed using the Omni Bead Ruptor Elite cooled using liquid nitrogen. RNA was quantified using the Qubit 4.0 Fluorometer (Thermo Fisher Scientific) with RIN scores determined using the Agilent 4200 TapeStation. All primers and probes used were “off-the-shelf” commercially available TaqMan Gene Expression Assays. Gene expression analyses were completed using the 2^−ΔΔCt^ method (Livak and Schmittgen, 2001), with the geometric mean of beta-2-microglobulin (B2M) and 18Sr as the normalization factor.

#### Skeletal muscle protein expression and nitrosylation measurements

Next, 5–10 mg of the right plantaris were lysed using RIPA buffer (Thermo Fisher Scientific, Cat. No. 89900) with a protease and phosphatase inhibitor cocktail (MilliporeSigma, Cat. No. PPC1010) using the Omni Bead Ruptor Elite cooled with liquid nitrogen. After homogenization, the lysates were rested on wet ice for 30 min, with 15 s of vigorous vortexing every 10 min. The lysates were then spun at 14,000 rcf for 15 min at 4°C, and the supernatant was collected. microBCA (Thermo Fisher Scientific, Cat. No. 23235) was used to determine the protein concentration. For the plantaris, 1.2 µg of protein was used for automated capillary electrophoresis (Jess, ProteinSimple), with a 1:50 primary antibody dilution used for all targets. Target protein expression was detected using the “Chemi” channel and normalized to the total protein content detected within the same capillary (i.e., no separate run was needed). Peaks were detected using the “dropped down” algorithm and quantified with the “total dynamic range” algorithm using ProteinSimple’s Compass for Simple Western software. All raw detection and analysis files for each protein are available in File S1 and can be accessed using the freely available Compass for Simple Western software.

3-nitrotyrosine was measured using 100 µg of protein from plantaris lysate mixed in assay diluent using the Cell Biolabs’ Nitrotyrosine Colorometric Kit (Cat. No. STA-305) following the manufacturer’s guidelines.

All supplies and reagents used in this series of experiments, alongside the vendor and catalog numbers, are given in Supplementary Table S1.

## Statistics

All statistical analyses were completed using GraphPad Prism (v10.1.1). All data for the T9 complete transection were analyzed using the “surgery x genotype” two-way mixed model ANOVAs as there was not sufficient statistical power to determine sex-specific differences. Single-endpoint data (e.g., muscle mass and molecular outcomes) for the T9 impact contusion studies were analyzed using two-way mixed model ANOVAs, with a three-way mixed model ANOVA “genotype x sex x time” used for the time series data (body mass change and behavioral tests). Both analysis strategies used Tukey’s multiple comparison post-testing when appropriate (denoted by “adj. p”), with statistical thresholds for meaningful differences set at *p* < 0.050. Unpaired (parametric) and Mann–Whitney (non-parametric) *t*-tests were used for two-group comparisons when appropriate, with statistical cutoffs for meaningful differences set at *p* < 0.050.

## Results

### T9 transection

#### Body and muscle mass

**FIGURE 1:**
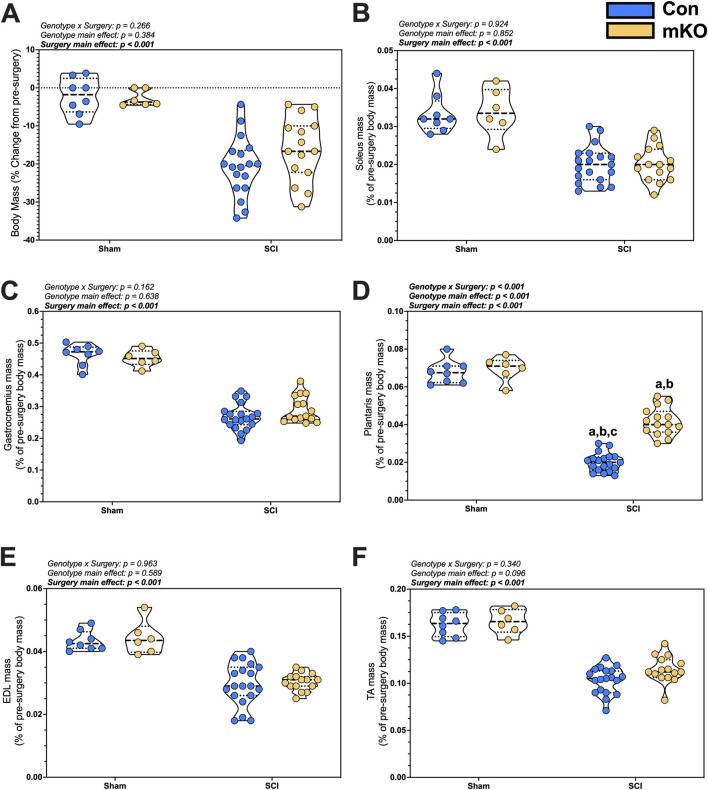
Changes in body and tissue mass 15 days after a complete transection SCI. **(A)** Percent change in body mass. Normalized wet tissue mass at the time of euthanasia for the **(B)** soleus, **(C)** gastrocnemius, **(D)** plantaris, **(E)** EDL, and **(F)** TA. Data are shown as violin plots with individual data points. Thin dashed lines represent quartiles, with the thick dashed line representing the mean. **(A)** = adj. *p* < 0.050 vs. Con-sham; **(B)** = adj. *p* < 0.050 vs. mKO-SCI; and **(C)** = adj. *p* < 0.050 vs. mKO-SCI. Group sizes: Con-sham (n = 8, 4M/4F), mKO-sham (n = 6, 3M/3F), Con-tSCI (n = 19, 10M/9F), and mKO-tSCI (n = 15, 4M/11F).

A main effect for greater body mass losses was observed in tSCI mice compared to sham mice (*p* < 0.001; Figure 1A). There was no significant effect of genotype on the body mass (Figure 1A).

There were main effects for reduced soleus and gastrocnemius mass in the tSCI mice compared to sham mice (both muscles *p* < 0.001; Figures 1B, C). There was a “genotype x surgery” interaction effect for the plantaris (*p* < 0.001; Figure 1D). Multiple comparison post-testing showed that both tSCI groups were reduced compared to both sham groups, with the mKO-tSCI group having greater mass than the Con-tSCI group (adj. *p* < 0.001). There were main effects for reduced mass in the EDL, TA, biceps, triceps, diaphragm, and heart in the tSCI mice compared to the sham mice (*p* < 0.001–0.010; Figures 1E, F; Supplementary Figures S1A–D), but there was no effect of genotype on the weights of these muscles.

#### mRNA expression

**FIGURE 2:**
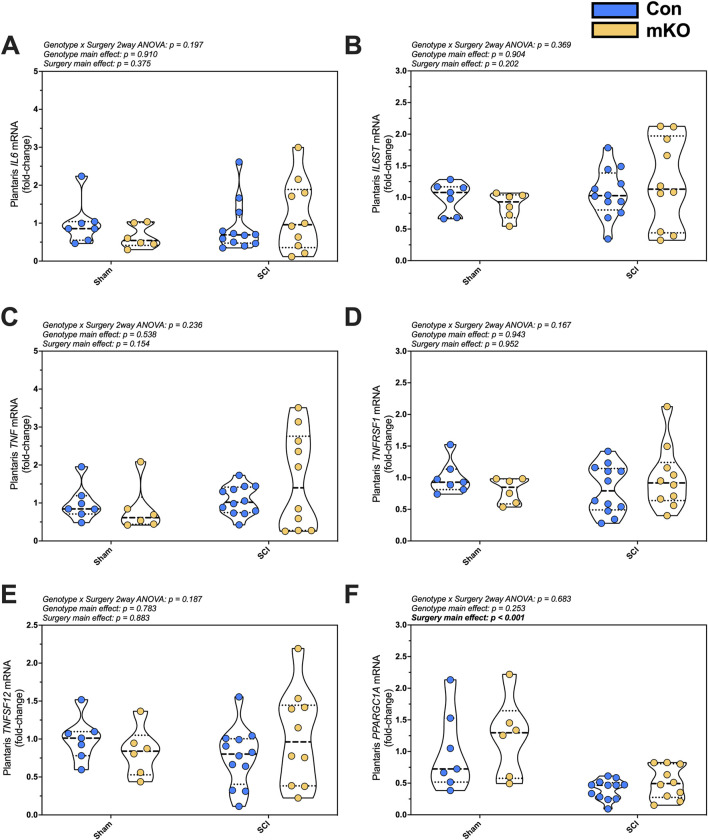
mRNA expression of key proinflammatory and mitochondrial markers in the plantaris after complete SCI. Expression of the cytokine **(A)** *IL6* and the signaling component of its receptor **(B)** *IL6ST* (encoding gp130). Gene expression of factors required for the activation of NF-κB signaling: **(C)** *TNF*, **(D)** *TNFRSF1*, and **(E)** *TNFSF12*. mRNA levels of the key mitochondrial biogenesis regulator **(F)** *PPARGC1A.* Data are shown as violin plots with individual data points. Thin dashed lines represent quartiles, with the thick dashed line representing the mean. Group sizes: Con-sham (n = 7, 4M/3F), mKO-sham (n = 6, 3M/3F), Con-tSCI (n = 12, 6M/6F), and mKO-tSCI (n = 10, 4M/6F).

Relative expression levels of the inflammatory factors *IL6, IL6ST, TNF, TNFRSF1*, *TNFSF12*, and *NKFBIA* were determined in plantaris mRNA but were not changed post-SCI (Figures 2A–E, respectively). There was a main effect for reduced expression in *PPARGC1A*, which encodes PGC1α following SCI (*p* < 0.001; Figure 2F). There were no group differences for the E3 ligase genes *FBOX32* (encoding MAFbx; Supplementary Figure S2A) and *TRIM63* (encoding MuRF1; Supplementary Figure S2B) or mitochondrial E3 ligase genes *MUL1* and *PARK2* (Supplementary Figures S2C–D, respectively). There were also no differences among groups for fast myosin expression [*MYH1* (type IIx), *MYH2* (encoding type IIa), or *MYH4* (encoding type IIb); Supplementary Figures S2E–G, respectively].

#### Protein expression

**FIGURE 3:**
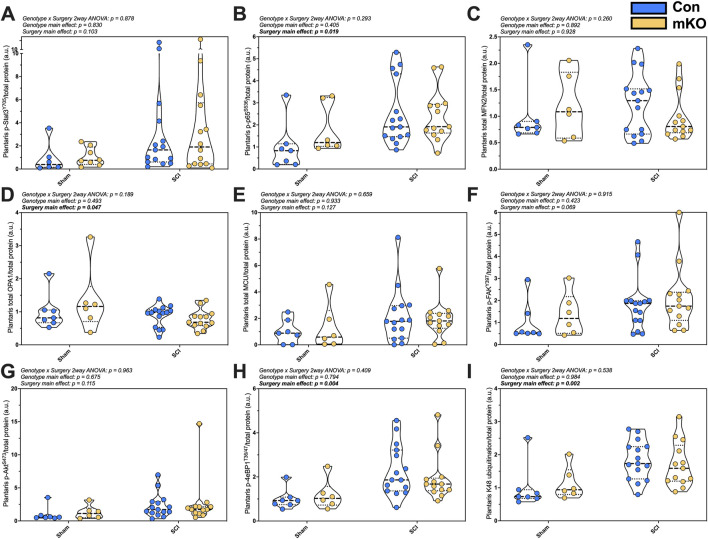
Plantaris whole-muscle protein expression following complete SCI. Phosphorylated protein expression for activated inflammasome markers **(A)** pSTAT3^Y705^ and **(B)** p-p65-NF-κB^S536^. Total protein expression of regulators of mitochondrial function: **(C)** mfn2, **(D)** OPA1, and **(E)** MCU. Phosphorylation of key activation sites for the mechanosensor **(F)** p-FAK^Y397^, the mTORC2 target **(G)** p-Akt^S473^, and downstream translational regulator **(H)** p-4eBP1^T36/47^. Lastly, **(I)** smear of K48-linked ubiquitin. Data are shown as violin plots with individual data points. Thin dashed lines represent quartiles, with the thick dashed line representing the mean. Group sizes: Con-sham (n = 7, 4M/3F), mKO-sham (n = 6, 3M/3F), Con-tSCI (n = 15, 8M/7F), and mKO-tSCI (n = 13, 4M/9F).

Markers of inflammasome activation, mitochondrial integrity, and hypertrophy were measured in the plantaris. There was no difference in p-Stat3^Y705^ following SCI (*p* = 0.103), but there were elevated levels of p-NFκB^S536^ in the SCI mice (*p* = 0.019; Figures 3A, B, respectively). There were no differences in the mitochondrial outer-membrane fusion protein Mfn2 (Figure 3C), but there was a main effect for the reduced inner-membrane fusion protein OPA1 expression post-SCI (*p* = 0.047; Figure 3D). There was no difference in the mitochondrial calcium channel (MCU) (Figure 3E) and no difference in the main activation site of the key mechanosensor p-FAK^Y397^ (Figure 3F). The expression of p-Akt^S473^ was not different among the groups (Figure 3G), but there was a main effect for elevated p-4eBP1^T37/46^ and K48 ubiquitination post-SCI (*p* = 0.004 and 0.002, respectively; Figures 3H, I). The representative images are provided in Supplementary Figure S3.

#### Muscle cytokine concentrations

A 10-plex cytokine ELISA was used to determine the concentrations of cytokines within the plantaris. Six cytokines were undetected or present at concentrations lower than needed for reliable measurements (IFN-γ, IL-1β, IL-4, IL-5, IL-6, and IL-12p70). There were no interactions or main-effect group differences for the four cytokines that were detectable: IL-2, IL-10, KC/GRO, and TNF-α (Supplementary Figures S4A–D).

#### Oxidative stress

There was no interaction effect (*p* = 0.290) or main effect of genotype (*p* = 0.081) or surgery (*p* = 0.907) in plantaris nitrotyrosine concentrations among the groups (Supplementary Figure S4E).

#### EDL contractile function

There was a main effect for elevated max relative twitch force in the SCI mice (*p* = 0.006; Supplementary Figure S5A), with no differences observed for time-to-peak tension or half-relaxation time (Supplementary Figures S5B, C). There was a “genotype x surgery” interaction effect for the fatigue index (*p* = 0.011; Supplementary Figure S5D). Multiple comparison post-testing showed that both tSCI groups had a greater fatigue index than both sham groups, with the mKO-tSCI group having a greater fatigue index than the Con-tSCI group (adj. *p* < 0.001–0.01). There was a main effect of genotype for max relative tetanic contraction (*p* = 0.008; Supplementary Figure S5E), with mKO mice having elevated force compared to Con mice. For the force–frequency contractile function, there was a “group x time” interaction, with multiple comparison post-testing showing multiple differences across the groups at each Hz.

#### T9 impact contusion

##### Body mass

**FIGURE 4:**
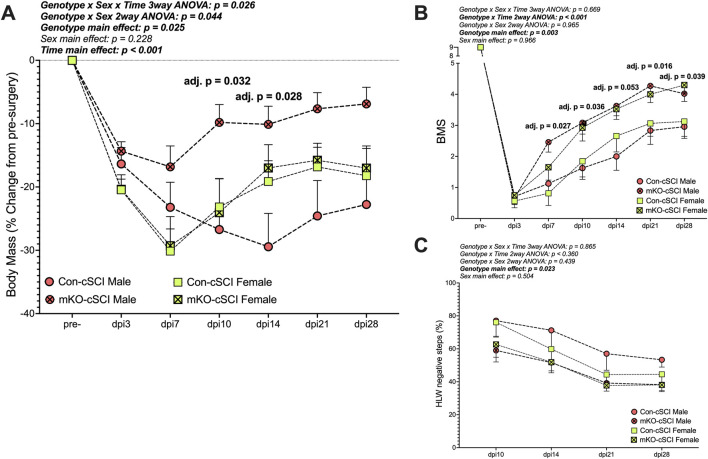
Muscle-restricted knockout improves functional outcomes after motor-incomplete SCI. **(A)** Body mass recovery of male and female knockout and genotype controls. Locomotor improvements over time for **(B)** the Basso Mouse Scale and **(C)** horizontal ladder walk tests. Data are shown as the mean +SEM with adjusted *p*-values for genotype^∗^time multiple comparison post-testing when applicable. Con-cSCI (n = 14, 8F/6M) and mKO-cSCI (n = 22, 10F/12M).

There was a “genotype x sex x time” interaction effect for the percent change in body mass (Figure 4A; *p* = 0.026). Follow-up two-way ANOVAs highlighted the “genotype x sex” interaction as statistically meaningful (*p* = 0.044). Simple-effect follow-up testing within time points demonstrated group differences in the males, with the mKO-cSCI mice having higher relative body mass (less weight loss) at dpi10 (adj. *p* = 0.032) and dpi14 (adj. *p* = 0.028) than Con-cSCI mice; body weights for mKO-cSCI mice appeared to remain higher than that of Con-cSCI mice until dpi28, although this effect did not reach our statistical threshold (adj. *p* = 0.101 at dpi14, and *p* = 0.080 at dpi28); there was no differential response for female mice.

#### Behavioral outcomes

There was a “genotype x time” interaction effect for BMS scores (Figure 4B; *p* < 0.001). Follow-up analyses showed greater BMS scores in mKO mice than in Con mice at dpi7 (adj. *p* = 0.027), dpi10 (adj. *p* = 0.036), dpi14 (adj. *p* = 0.053), dpi21 (adj. *p* = 0.016), and dpi28 (adj. *p* = 0.039). For HLW testing, no interaction effects were detected, but there was a main effect of genotype, with mKO mice having a reduced percentage of negative step outcomes compared to Con mice (*p* = 0.023; Figure 4C).

#### Tissue mass

**FIGURE 5:**
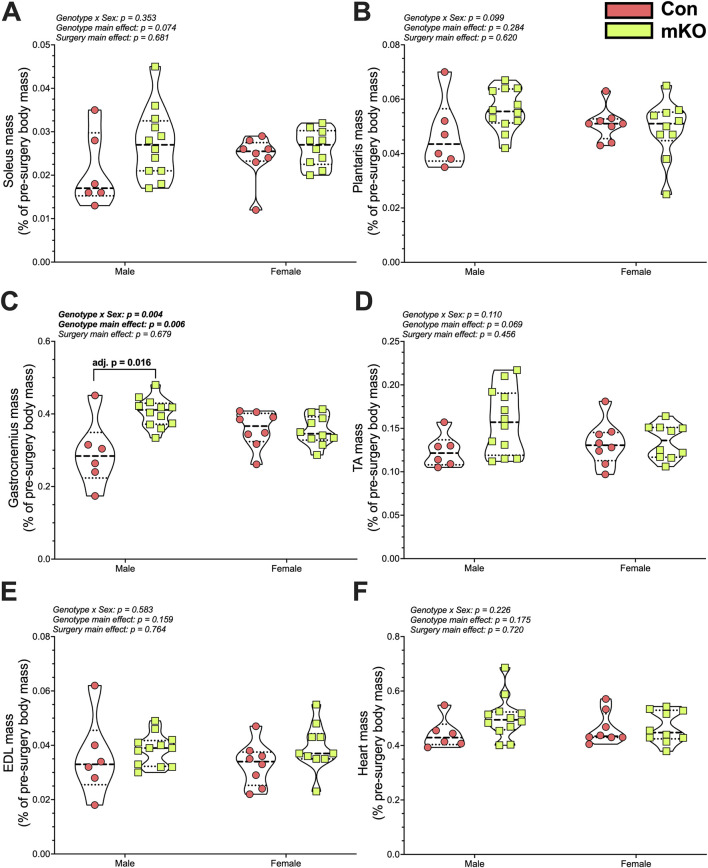
Wet tissue mass 28 days after motor-incomplete SCI. Excised and normalized wet tissue mases of the principal hind limb muscles: **(A)** soleus, **(B)** plantaris, **(C)** gastrocnemius, **(D)** TA, and **(E)** EDL. **(F)** Heart mass following cardiac puncture and exsanguination. Data are shown as violin plots with individual data points. Thin dashed lines represent quartiles, with the thick dashed line representing the mean. Con-cSCI (n = 14, 8F/6M) and mKO-cSCI (n = 22, 10F/12M). Data are shown as violin plots with individual data points. Thin dashed lines represent quartiles, with the thick dashed line representing the mean. Con-cSCI (n = 14, 8F/6M) and mKO-cSCI.

There were no differences in soleus or plantaris masses among the groups (Figures 5A, B). There was a “genotype x sex” interaction for the gastrocnemius, with follow-up testing showing greater mass in the male mKO-cSCI mice than in the male Con-SCI group (Figure 5C; adj. *p* = 0.001). There were no differences noted for the TA, EDL, or heart mass among the groups (Figures 5D–F).

#### Serum cytokine

**FIGURE 6:**
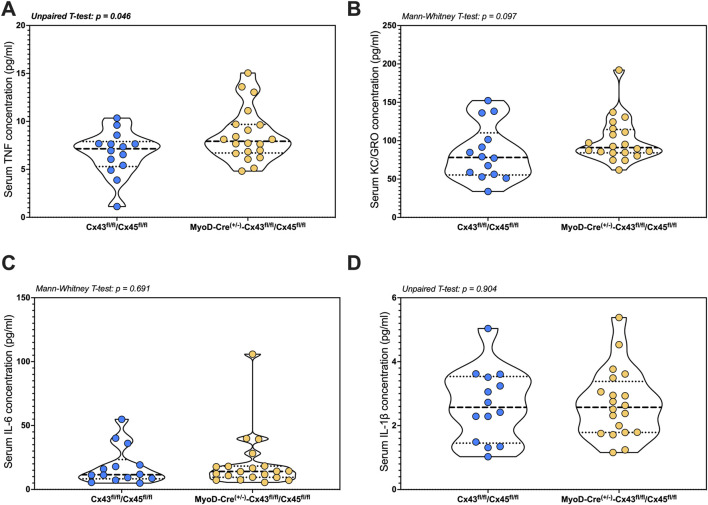
Circulating markers of inflammation following SCI between genotype controls and muscle-restricted knockouts. Serum concentrations for cytokines that were detected in the quantifiable range: **(A)** TNF, **(B)** KC/GRO, **(C)** IL-6, and **(D)** IL-1β. Data are shown as violin plots with individual data points. Thin dashed lines represent quartiles, with the thick dashed line representing the mean. Con-cSCI (n = 14, 8F/6M) and mKO-cSCI (n = 20, 19F/11M).

IL-4 was either undetectable or present at levels below those required for reliable quantification. Serum TNF-α was higher in the mKO-cSCI mice than that in Con-cSCI mice (*p* = 0.046; Figure 6A). There was no difference in serum levels between mKO-cSCI and Con-cSCI mice for KC/GRO, IL-6, and IL-1β (Figures 6B–D) or for IL-2, IL-5, IL-10, and IL-12p70 (Supplementary Figures S6A–D).

#### Spinal cord histology

**FIGURE 7:**
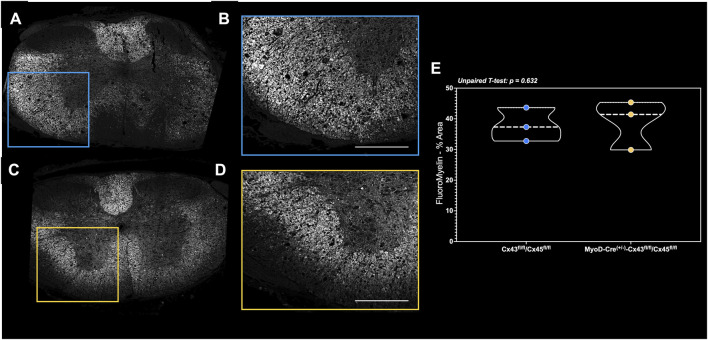
FluoroMyelin staining of perfusion-fixed spinal cords in a subset of mice. Spinal cord cross-sections of the **(A)** complete lesion FluoroMyelin stain and **(B)** quantified inset for the Con-cSCI mice. Similarly, **(C)** complete lesion FluoroMyelin stain and **(D)** quantified inset for the mKO-cSCI mice. **(E)** Quantification of FluoroMyelin staining as the percent area of inset. Data are shown as violin plots with individual data points. Thin dashed lines represent quartiles, with the thick dashed line representing the mean. Con-cSCI (n = 3, 2F/1M) and mKO-cSCI (n = 3, 2F/1M). Scale bar = 500 µm.

In the small subset of fixation-perfused animals, there were no differences in the markers of spinal cord integrity around the lesion site as the white and gray matter volume was similar between the groups (Figures 7A–E).

## Discussion

The goal of this study was to determine the biological significance of our previously reported findings that complete SCI results in the reappearance of sarcolemmal expression of multiple CxHC (Cx39/43/45) in rat gastrocnemius muscle (Cea et al., 2013). To accomplish this objective, we tested whether muscle-restricted knockout of Cx43/45, which has been demonstrated to be effective at reducing paralysis-induced atrophy following peripheral denervation, altered skeletal muscle outcomes in two different models of SCI. Because this model differs from atrophy following peripheral denervation (e.g., sciatic or peroneal nerve transection) in that the lower motor neuron is intact with some reflex activation remaining, a complete spinal cord transection was used to understand how Cx43/45 contributed to changes in skeletal muscle. We also tested whether muscle-restricted Cx43/45 knockout affects sensorimotor function following an impact contusion-induced motor-incomplete SCI. Our data show that the muscle-restricted knockout of Cx43/45 did not protect body mass and muscle mass or alter molecular outcomes after complete spinal cord transection, but such a model resulted in greater and faster functional recovery in motor-incomplete contusion SCI. These data highlight Cx43/45 as a potential regulator of motor unit function in motor-incomplete SCI.

### CxHC and muscle atrophy and function after tSCI

Transgenic and pharmaceutical approaches have demonstrated that both *in vivo* and *in vitro* knockout or inhibition of Cx43 and Cx45 is beneficial for muscle health and function by preventing excess intracellular calcium accumulation with resultant inflammasome activation, along with other cellular processes, in a variety of preclinical disuse and pathological models (Cea et al., 2013; Cisterna et al., 2020; Cea et al., 2019; Cea et al., 2023; Cisterna et al., 2016; Cea et al., 2016). The role of CxHCs in the muscle atrophy process following SCI is unknown outside their elevated sarcolemmal expression, as noted above. Our data convincingly show that muscle-restricted knockout of Cx43/45 does not protect against the loss of body mass or muscle mass 15 days after a complete SCI, with the exception being attenuated loss of plantaris mass. The lack of preservation of muscle and body mass highlights the established difficulty of protecting these outcomes in motor-complete SCI models (Otzel et al., 2021) and further suggests that motor-complete SCI is a multifactorial process resulting in muscle atrophy.

The explanation for the selective effect of mKO on the plantaris muscle is not well understood. *De novo* sarcolemmal expression of Cx43 and Cx45 occurs preferentially in type-II fibers, such as the plantaris, and results in intracellular calcium accumulation, a key molecular signal that activates the muscle inflammasome (Cea et al., 2013) and initiates mitochondrial dysfunction and oxidative stress (González-Jamett et al., 2022). We measured molecular markers of these domains and could not find a unique genotype profile. The mRNA expression of key pro-inflammatory markers and their receptors was not altered among groups. We did observe an elevation in p65-NFκB activation and mean changes in Stat3 activation after SCI, suggesting potential pro-inflammatory signaling, but we observed no effect of the genetic knockout. Furthermore, markers of mitochondrial integrity were not different among the groups, with the exception of SCI-induced reduction in mRNA expression of *PPGCA1A*, the gene encoding the mitochondrial regulator PGC1α, which we have reported previously (Graham et al., 2022). Although not reaching statistical thresholds, there was a distinct SCI-induced elevation in protein tyrosine nitrosylation that was prevented in mKO-tSCI plantaris muscle, consistent with the observed reduced atrophy, suggesting the potential for CxHCs to prevent cellular stress and excess protein nitrosylation. The markers of protein atrophy, such as gene expression of the major atrophy-associated E3 ligases, protein synthesis signaling, and protein ubiquitination, were not altered by our mKO. Thus, it appears that the plantaris was uniquely affected with no clear molecular signature. The responsible molecular mechanisms and effects on muscle metabolic and contractile function remain to be defined.

### Effects of mKO in a cSCI model

Our major finding was the relevance of skeletal muscle Cx43/45 in hindlimb locomotor function after a motor-incomplete cSCI. BMS scoring, which tests observations of hindlimb movement and stepping based on simple criteria (Basso et al., 2006), revealed that mKO mice recovered faster and to a greater degree than Con-cSCI mice. When using HLW, which quantifies positive steps (e.g., correct plantar placement) and negative steps (e.g., slipping off of a horizontal rung) to measure more fine motor function (Cummings et al., 2007), there was a clear main effect for the reduction in the percent of negative step outcomes in the mKO mice compared to Con-cSCI mice. mKO mice did not have greater white matter sparing based on FluoroMyelin staining. One interpretation of this result is that the *de novo* sarcolemmal expression of CxHC in the Con-cSCI mice likely acts on the motor unit to degrade NMJ integrity. This is supported by a previous work showing that sarcolemmal CxHC largely prevents innervation using *in vitro* co-cultures of isolated myofibers and primary dorsal root ganglia. When sarcolemmal Cx43/45 is reduced by targeting mRNA using morpholinos or *via* transgenic approaches using muscle-restricted knockouts, innervation is ∼5–6-fold greater (∼5–10% to 50%–60%) (Cisterna et al., 2020). Other explanations include mKO mice having greater synaptic strength of residual neural circuits to support sensorimotor function and greater numbers of relay circuits that convey signals between the brain and hind limbs. Our current data do not provide sufficient evidence to distinguish between these possibilities.

In contrast to the tSCI model, we noted distinct genotype differences in the cSCI model. Regarding body mass, female mice in both the mKO and Con groups and male mKO mice began to recover 7 days post-SCI, which is similar to our general timeline using both contusion and transection models (Toro et al., 2023; Graham et al., 2015; Graham et al., 2022). However, male Con mice continued losing body mass up to 14 days. By the end of our 28-day experiment, mKO males had the greatest relative preservation in body mass, while the Con males had the poorest. Reduced food intake and excess body fat loss are logical explanations for our finding, but neither of these possibilites were investigated in this report; thus, confirmatory studies in this SCI genotype model that investigate these endpoints are required.

Data generated from our contusion mKO mice are consistent with our reported effects of orally administered boldine, a noted CxHC inhibitor, on sensorimotor function. Boldine has been shown to recapitulate multiple effects on muscle health and function compared to muscle-restricted CxHC knockout models *in vivo* and *in vitro* (Cea et al., 2019; Cea et al., 2023; Cea et al., 2020)*.* In prior work, both male and female mice that received 50 mg/kg/d boldine had greater BMS scores and a reduced proportion of negative HLW steps than vehicle-treated mice across 28 days^27^. However, the magnitude of difference between the experimental and control animals was larger in the boldine study. This difference may be explained by the systemic administration of boldine with this agent’s other beneficial effects on the central nervous system, as our group observed that boldine administration reduced white sparing and glial cell activation at the lesion site, along with neurorecovery signatures in spinal cord tissue directly below the lesion (Toro et al., 2023). In addition to CxHC antagonism, boldine blocks other channel proteins, such as pannexins and P2XR7, which represent additional therapeutic targets independent of CxHCs. The available evidence suggests that peripheral CxHCs play a role in reducing the sensorimotor function in motor-incomplete SCI. Further investigation is needed to address the precise mechanisms responsible for our findings, such as alterations in the muscle secretome, to address the gap in our knowledge.

When analyzing sex-based variation, it is important to note that the recovery of body mass post-SCI showed differences between male and female mice. These results suggest sex-specific differences in response to the injury and subsequent recovery processes, with male mKO mice having better overall outcomes in terms of body mass retention than their female counterparts. However, our mixed-model approach did not highlight sex as a main effect in a meaningful manner, likely due to being underpowered to detect this difference explicitly. Thus, there may be a biological mechanism(s) underlying recovery after SCI that is influenced by sex. Future studies should aim to further investigate sex-specific molecular and physiological responses to improve targeted treatments.

## Conclusion

The hypothesis of this study proposed that muscle-restricted knockout of Cx43/45 would mitigate muscle atrophy and improve motor function outcomes following SCI, particularly in models where some degree of neural activation remained, such as after an incomplete SCI. The results partially support this hypothesis, especially in the motor-incomplete cSCI model, where mKO of Cx43/45 led to faster and more complete functional recovery, as demonstrated by the improved hind limb locomotor function and reduced proportion of negative stepping outcomes. However, the hypothesis did not align with the results in the motor-complete SCI (tSCI) model. In this case, the knockout of Cx43/45 neither protected against muscle or body mass loss nor showed significant molecular changes, aside from some selective muscle effects in the plantaris. This suggests that while Cx43/45 may play a role in motor unit function preservation in motor-incomplete SCI, it does not significantly influence muscle atrophy in more severe injury models where the motor neuron is entirely severed. Thus, the results indicate that Cx43/45 plays a more complex and context-dependent role than initially hypothesized, with its benefits primarily evident in conditions where some motor function remains, highlighting the importance of motor unit integrity for muscle preservation and recovery.

There was no significant effect observed following mKO of Cx43/45 15 days after a complete spinal cord transection at the whole-body, individual muscle (with the exception of the plantaris), and molecular level. However, in both male and female mice across 28 days following motor-incomplete contusion SCI, the mKO model resulted in greater and more rapid functional recovery, without any clear effects on the spared white matter. There are several considerations and limitations to our studies. The post-operative death rate following cSCI was higher than typically observed in such studies without a discernable explanation. Cx43^(fl/fl)^/Cx45^(fl/fl)^ was generated on a C57BL6 background, with the MyoD-Cre^(+/−)^ mice on an FVB background, which is a common cross not expected to result in any unexpected behavior, and this strain crossing has even been suggested as a better model for neurobehavioral outcomes, including locomotor tests such as the rotarod Sloin et al., 2022. We also did not record food intake or activity levels in the cSCI mice, which would have helped better understand the distinct sex and genotype effect on body mass and whether the improvements in locomotor function were related to cage activity, food intake, or other general markers of improved systemic health and function. Lastly, we did not investigate any molecular outcomes in the muscle from cSCI mice. The effects of increased muscle loading of mKO mice as the result of their improved sensorimotor function and associated increased capacity to walk may be hypothesized to be of greater consequence than the molecular changes induced by the mKO model alone, which may potentially confound the interpretation of the results. Further study is required to better understand the mechanisms by which sarcolemmal Cx43/45 contributes to tissue injury and subsequent functional recovery after traumatic motor-incomplete SCI.

## Data Availability

The original contributions presented in the study are included in the article/Supplementary Material, further inquiries can be directed to the corresponding authors.

## Supplementary material

SUPPLEMENTARY FIGURE S1 Wet weights following 15 days of complete SCI for the **(A)** triceps, **(B)** biceps, and **(C)** exsanguinated heart. Data are shown as violin plots with individual data points. Thin dashed lines represent quartiles, with the thick dashed line representing the mean. Group sizes: Con-sham (n = 8, 4M/4F), mKO-sham (n = 6, 3M/3F), Con-tSCI (n = 14–19, 7–8M/7–10F), and mKO-tSCI (n = 14–15, 4M/10–11F).

SUPPLEMENTARY FIGURE S2 mRNA expression assays for atrophy markers **(A)** *FBOX32* (encoding MAFxb) and **(B)** *TRIM63* (encoding MuRF1); mitophagy markers **(C)** *MUL1* and **(D)** *PARK2*; and myosin heavy chain **(E)** *MYH1* (encoding myosin IIx), **(F)** *MYH2* (encoding myosin IIa), and **(G)** *MYH4* (encoding myosin IIb). Data are shown as violin plots with individual data points. Thin dashed lines represent quartiles, with the thick dashed line representing the mean. Group sizes: Con-sham (n = 7, 4M/3F), mKO-sham (n = 6, 3M/3F), Con-cSCI (n = 12, 6M/6F), and mKO-cSCI (n = 10, 4M/6F).

SUPPLEMENTARY FIGURE S3 Representative immunodetected images for each reported protein.

SUPPLEMENTARY FIGURE S4 Protein assays from whole plantaris lysate 28 days after a motor-incomplete SCI. Multiplex cytokine concentrations for **(A)** IL-2, **(B)** IL-10, **(C)** KC/GRO, and **(D)** TNF. **(E)** Plantaris protein nitrotyrosine concentrations. Data are shown as violin plots with individual data points. Thin dashed lines represent quartiles, with the thick dashed line representing the mean. For **(A–D)**, Con-tSCI (n = 9, 4M/5F) and mKO-tSCI (n = 8, 4M/4F). For (E) Group sizes: Con-sham (n = 7, 4M/3F), mKO-sham (n = 6, 3M/3F), Con-tSCI (n = 12, 6M/6F), and mKO-tSCI (n = 10, 4M/6F).

SUPPLEMENTARY FIGURE S5
*Ex vivo* contractile function of the EDL muscle. Twitch contraction outcomes for **(A)** maximal relative EDL twitch force, **(B)** time-to-peak tension, and **(C)** half relaxation time. Tetanic contraction outcomes for **(D)** fatigue index. **(E)** Max tetanic force and **(G)** force/frequency relationship. Panels **(A-E)** are shown as violin plots with individual data points. Thin dashed lines represent quartiles, with the thick dashed line representing the mean. Panel **(F)** is shown as the mean ± SEM. (a) = adj. *p* < 0.050 vs. Con-sham; (b) = adj. *p* < 0.050 vs. mKO-sham; (c) = adj. *p* < 0.050 vs. Con-SCI. Con-sham (n = 8, 4M/4F), mKO-sham (n = 6, 3M/3F), Con-tSCI (n = 14, 7M/7F), and mKO-tSCI (n = 14, 4M/10F).

SUPPLEMENTARY FIGURE S6 Serum proinflammatory cytokine concentrations 28 days after a motor-incomplete SCI. Con and mKO levels were determined for **(A)** IL-2, **(B)** IL-5, **(C)** IL-10, and **(D)** IL-12p70. Data are shown as violin plots with individual data points. Thin dashed lines represent quartiles, with the thick dashed line representing the mean. Con-cSCI (n = 14, 7M/7F) and mKO-tSCI (n = 20, 10M/10F).
