# Correction: Muscle-restricted knockout of connexin 43 and connexin 45 accelerates and improves locomotor recovery after contusion spinal cord injury

**DOI:** 10.3389/fphys.2025.1711889

**Published:** 2025-10-13

**Authors:** Carlos A. Toro, Rita De Gasperi, Katherine Vanselow, Lauren Harlow, Kaitlin Johnson, Abdurrahman Aslan, William A. Bauman, Christopher P. Cardozo, Zachary A. Graham

**Affiliations:** ^1^ Spinal Cord Damage Research Center, Bronx, NY, United States; ^2^ Department of Medicine, Icahn School of Medicine at Mount Sinai, New York, NY, United States; ^3^ Department of Phychiatry, Icahn School of Medicine at Mount Sinai, New York, NY, United States; ^4^ The Friedman Brain Institute, Icahn School of Medicine at Mount Sinai, New York, NY, United States; ^5^ Healthspan, Resilience and Performance, Florida Institute for Human and Machine Cognition, Pensacola, FL, United States; ^6^ Research Service, Birmingham VAHCS, Birmingham, AL, United States; ^7^ Department of Cell, Developmental, and Integrative Biology, University of Alabama at Birmingham, Birmingham, AL, United States

**Keywords:** spinal cord injury, Cx43, skeletal muscle-restricted knockout, Cx45, contusion spinal cord injury, transection spinal cord injury, recovery of function

In the published article, the funder NYS SCIRB, C38329GG to CC was erroneously omitted.

The corrected funding statement appears below.

The author(s) declare that financial support was received for the research and/or publication of this article. This study was funded by the Department of Veterans Affairs Office of Research and Development R&D Service CDA-2 grant (1IK2RX002781 to ZAG) and Center grant (5I50RX002020 to WAB). NYS SCIRB, C38329GG to CC.

The original version of this article has been updated.

