# Supplementary figures and images for: Muscle-restricted knockout of connexin 43 and connexin 45 accelerates and improves locomotor recovery after contusion spinal cord injury
Source: Front Physiol. 2024 Oct 25;15:1486691. doi: 10.3389/fphys.2024.1486691 (PMC11543431; doi:10.3389/fphys.2024.1486691)

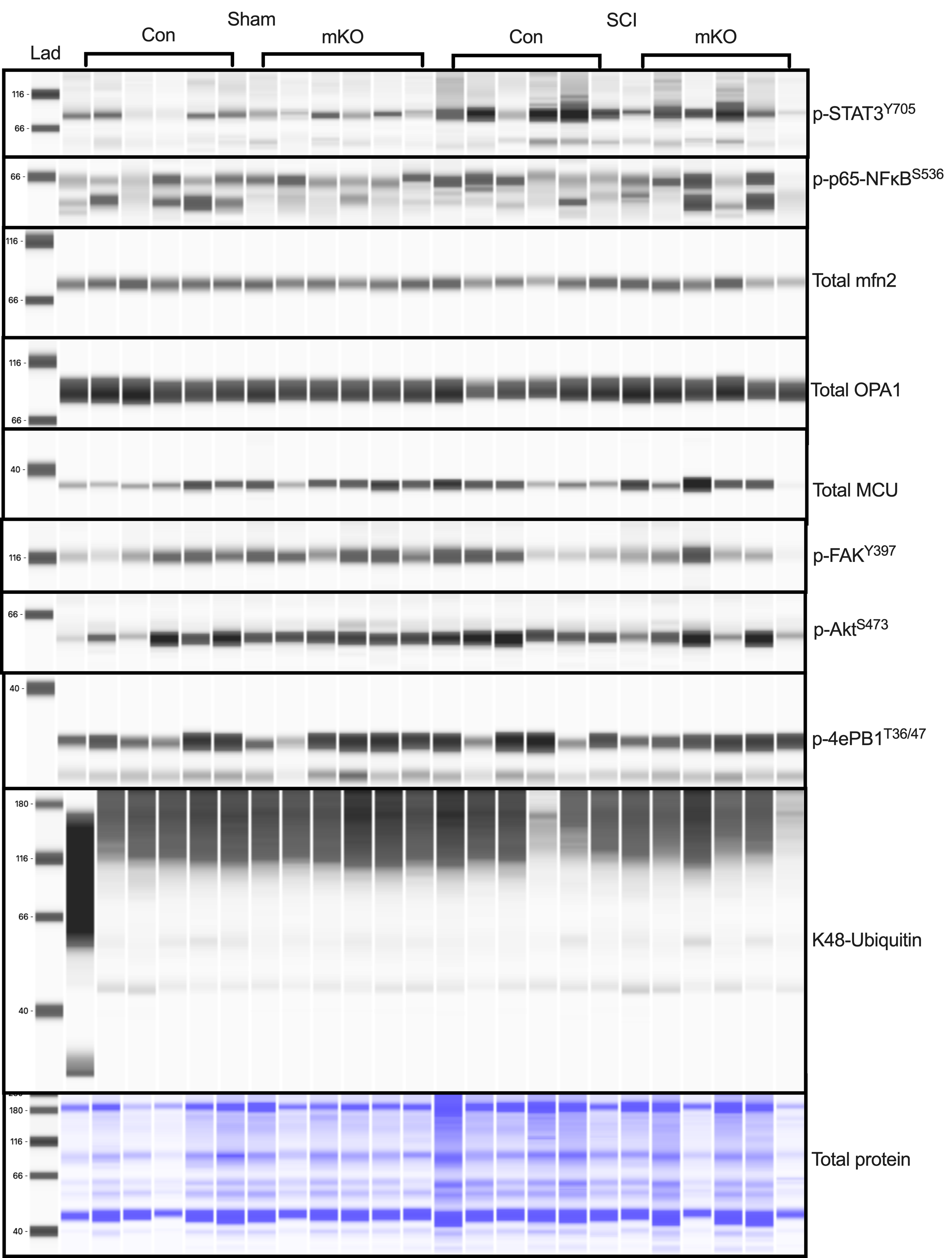

Supplement: Supplementary file 1 [file Image3.TIFF]

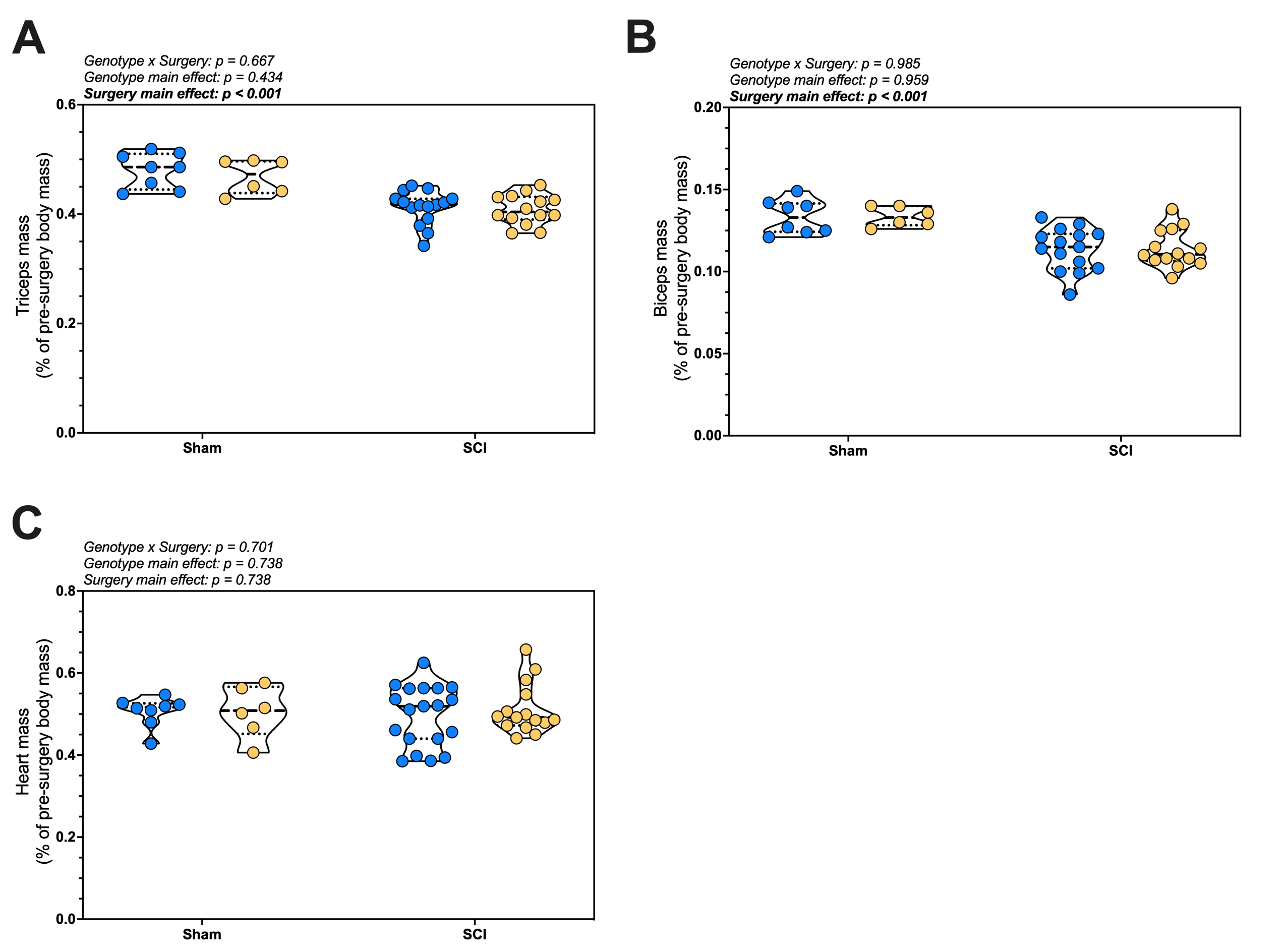

Supplement: Supplementary file 2 [file Image1.TIFF]

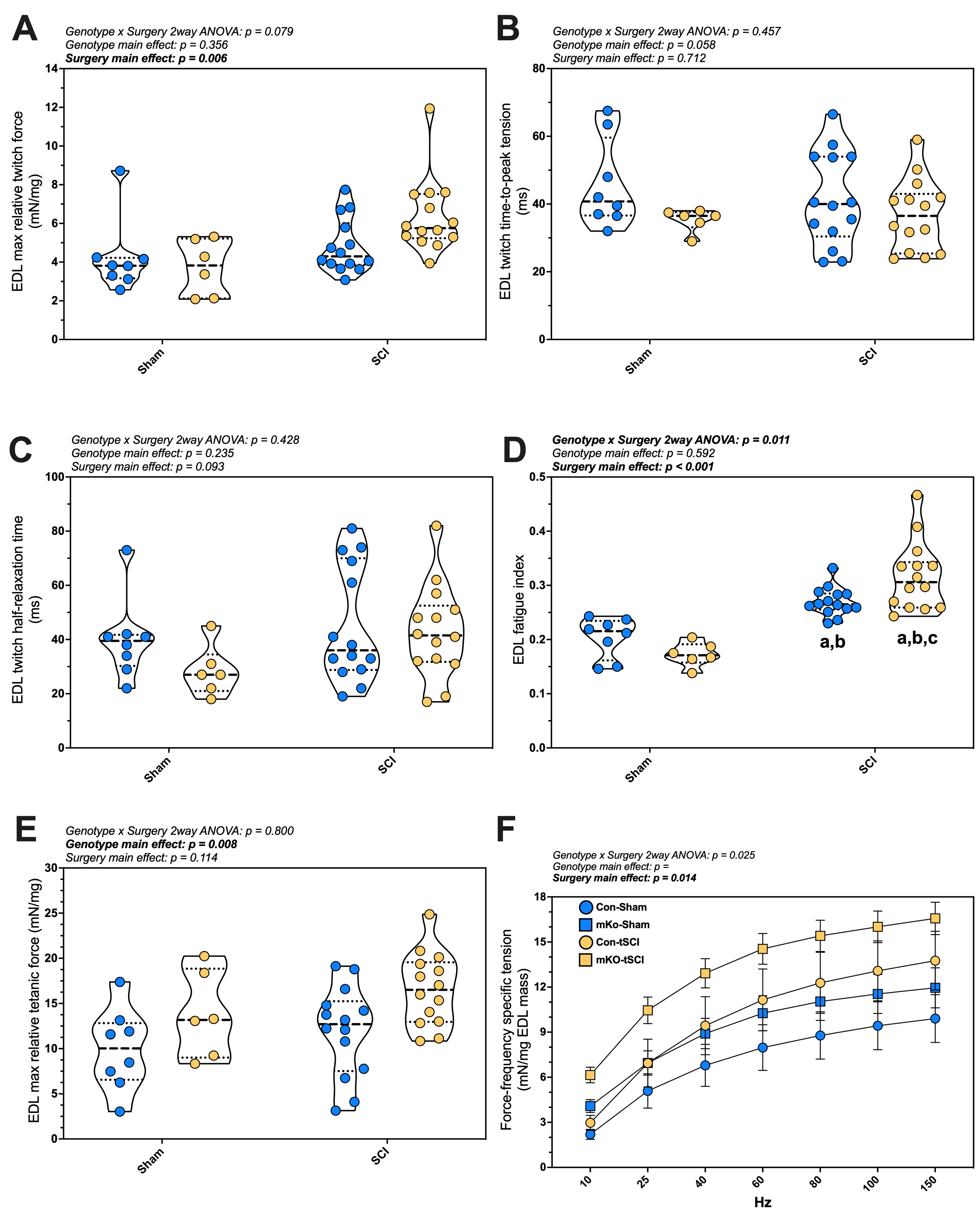

Supplement: Supplementary file 3 [file Image5.TIFF]

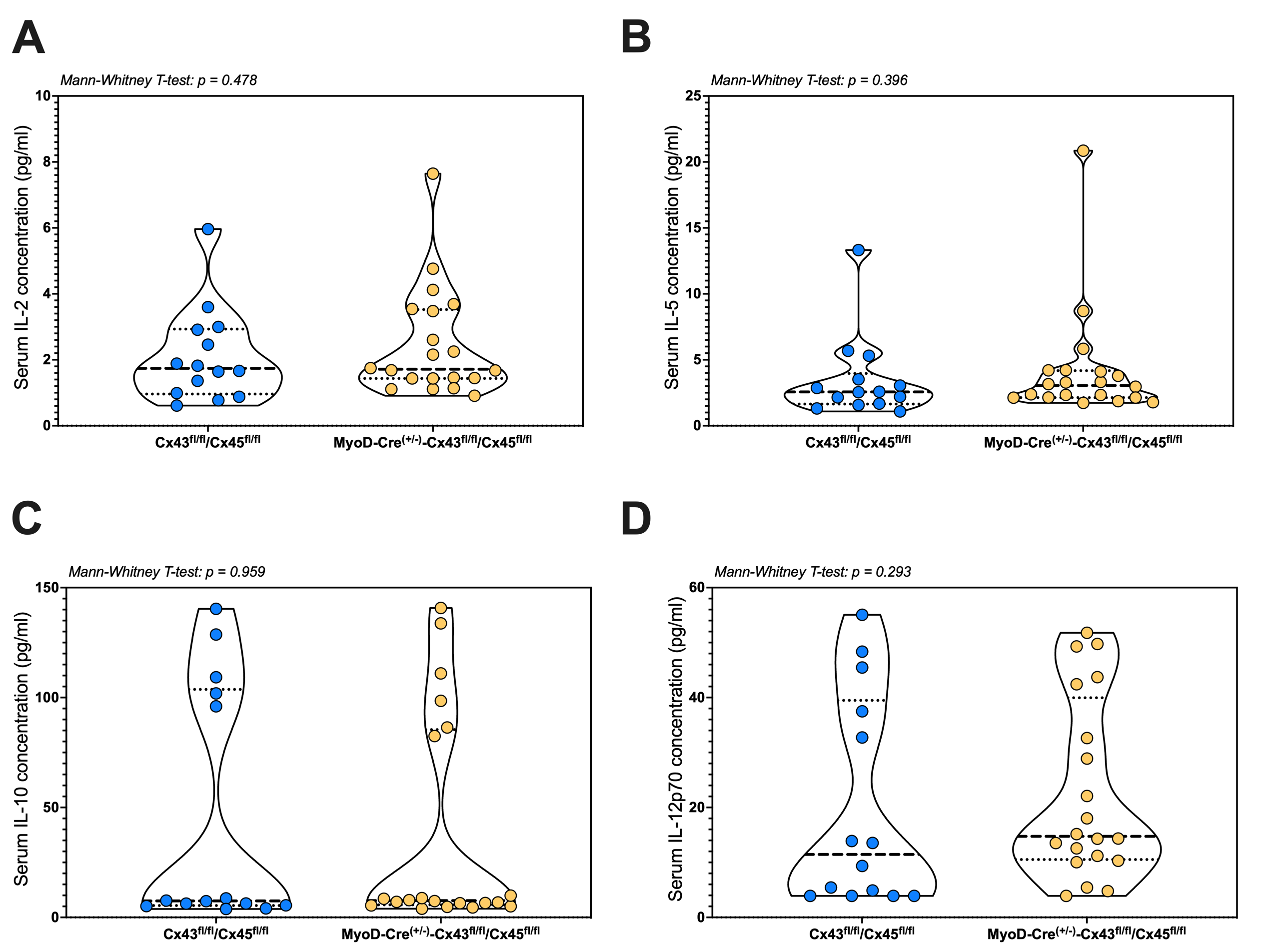

Supplement: Supplementary file 4 [file Image6.TIFF]

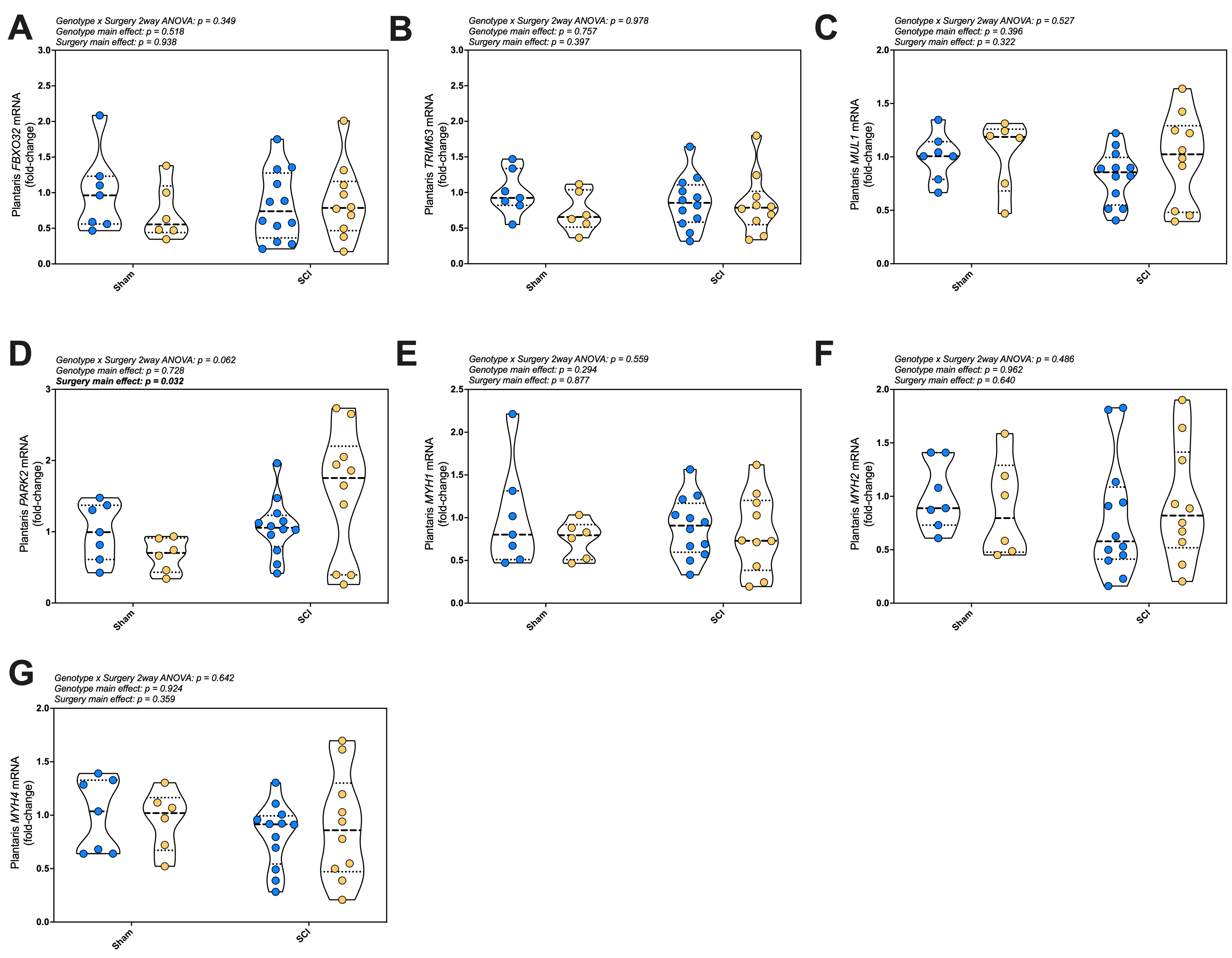

Supplement: Supplementary file 5 [file Image2.TIFF]

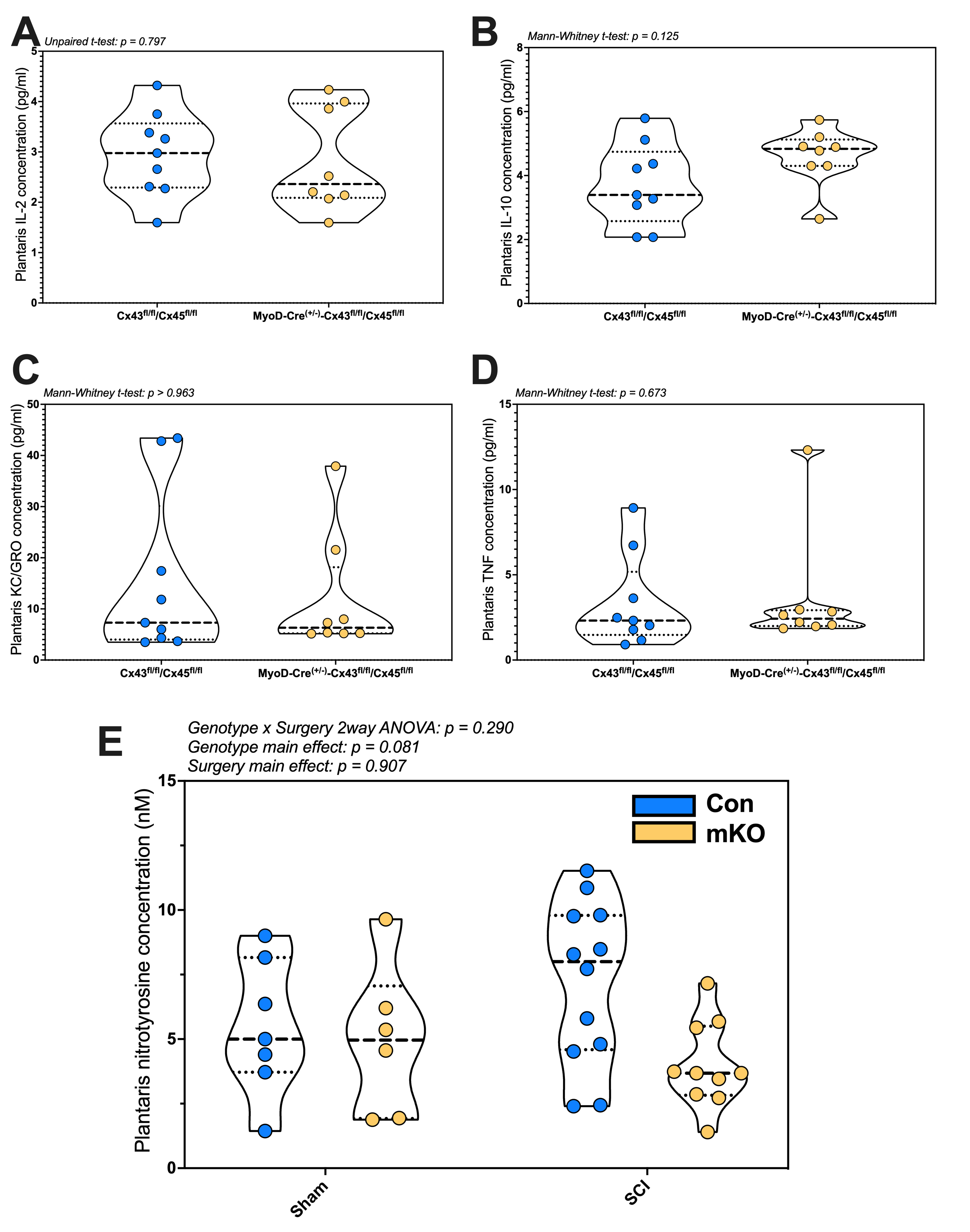

Supplement: Supplementary file 6 [file Image4.TIFF]
